# Choice of Pole and Ski Lengths Among Elite Cross-Country Skiers: The Influence of Sex and Performance Level

**DOI:** 10.3389/fspor.2021.654864

**Published:** 2021-04-22

**Authors:** Per-Øyvind Torvik, Roland van den Tillaar, Øyvind Sandbakk

**Affiliations:** ^1^Department of Sports Sciences and Physical Education, Nord University, Levanger, Norway; ^2^Department of Neuromedicine and Movement Science, Centre for Elite Sports Research, Faculty of Medicine and Health Sciences, Norwegian University of Science and Technology, Trondheim, Norway

**Keywords:** cross-country skiing, ski characteristics, pole characteristics, performance, gender differences, XC skiing

## Abstract

Cross-country (XC) skiers employ whole-body exercise to generate speed through poles and skis. The choice of optimal pole and ski lengths are therefore of high importance. The aim of this study was to document pole and ski lengths among elite male and female cross-country skiers in the classical and skating styles and to investigate sex differences in body-height-normalized pole and ski lengths. Our secondary purpose was to correlate body-height-normalized pole and ski lengths with performance level within both sexes. In total, Norwegian men and women (*n* = 87 and 36, respectively), participating in the Norwegian XC championship 2020, were investigated. Most athletes used poles close to the length allowed by the International Ski Federation (FIS) in the classical style among both sexes, with men using slightly longer poles than women (*p* < 0.05). Body-height-normalized pole lengths in skating were similar in men and women (around 90% of body height). Women used relatively longer ski lengths than men in both styles (*p* < 0.05). Women showed moderate correlations (*r* = 0.43, *p* < 0.05) between body-height-normalized pole lengths and sprint performance. Male and female cross-country skiers use as long classical ski poles as possible within the current regulations, while they use skating poles similar to recommendations given by the industry. The fact that men use longer body-height-normalized poles than women, where there is a correlation between pole length and sprint performance, indicate that faster women are able to better utilize the potential of using longer poles when double-poling. However, while women use relatively longer skis than men, no correlation with performance occurred for any of the sexes.

## Introduction

Cross-country skiing is a winter endurance sport, performed while gliding over snow-covered hilly terrain using different sub-techniques of the classical and skating styles (Sandbakk and Holmberg, 2017). During this locomotion, skiers engage large muscle groups of the upper and lower limbs to generate and transfer power through poles and skis into the snow, thereby accelerating the center of mass forward (Holmberg, 2015). Consequently, an ongoing development of poles and skis aims to optimize this transfer of force, and at the same time, minimize energy dissipation through reduced friction and air drag. Accordingly, the development of performance in cross-country skiing is aided by concurrent improvements in skier capacity and equipment development (Pellegrini et al., 2018).

Although skis and poles are the main generators of propulsion in cross-country skiing, their characteristics are sparsely examined. However, harder ski tracks, better endurance-trained upper bodies of skiers (Stöggl and Holmberg, 2011), along with the introduction of sprint skiing and the professionalization of long-distance cross-country skiing have motivated skiers to experiment with longer pole lengths in the classical style. Indeed, several recent studies have indicated that double-poling can be done more effectively by employing longer poles (Losnegard et al., 2017a,b), with similar advantages recently found in skating (Torvik et al., 2019). In order to prevent skiers from using the double-poling technique exclusively in the classical style, the International Ski Federation (FIS) have limited pole lengths to <83% of body height while wearing ski boots (FIS, 2020). In skating, the standard pole recommendations are ~20 cm below body height but FIS regulations do not allow poles to exceed an athlete's body height (FIS, 2020).

The guidelines for selecting ski lengths in classic and skating styles seem almost unchanged since the late 1980's, with typical recommendations being ~10–15 cm and ~25 cm above body height for skating and classic skis, respectively. While several previous studies have examined chamber height, ski stiffness, and grinding structures (Breitschädel, 2012), no studies have tested the effects of different ski lengths on performance in cross-country skiing. Breitschädel (2012) reported an average classical ski length in the Norwegian national team of 206 cm, with 8 cm shorter skis among women. That investigation did not evaluate ski length related to body height but reported an average of ~20% longer nominal contact area (the ski length minus the kick wax area) between the ski and the snow among women (Breitschädel, 2012), indicating that women are using longer skis in relation to their body height.

Although some experimental studies have reported advantages of using longer poles in both the classical (Carlsen et al., 2018; Torvik et al., 2021a) and skating techniques (Torvik et al., 2019), no systematic report on elite skiers' employment of pole and ski lengths currently exists. In this context, possible sex and performance-level differences in body-height-normalized pole lengths are of high interest. Therefore, this study's primary purposes were to document pole and ski lengths among elite male and female cross-country skiers in the classical and skating styles and to investigate sex differences in body-height-normalized pole and ski lengths. Our secondary purpose was to correlate body-height-normalized pole and ski lengths with skiing performance within both sexes. Our main hypothesis was that men would use longer poles than women and that women would use longer skis, in both cases when poles or skis where normalized for body height.

## Methods

### Participants

Eighty-seven male and 36 female cross-country skiers who participated in the 2020 Norwegian Championships were included in this study. Inclusion criteria were that skiers competed in both the classical and skating styles, completed the questionnaire and systematically reported training in a diary. The level of male and female skiers was relatively evenly divided, from the best performers with 0 FIS points to the lowest-ranked skiers having 400 FIS points.

The Regional Committee for Medical and Health Research Ethics waives the requirement for ethical approval for such studies. Therefore, the ethics of the study was carried out according to the institutional requirements and approval for data security was obtained from the Norwegian Centre for Research Data. Prior to the data collection, all participants provided written and informed consent to take part in the study voluntarily. The participants were informed that they could withdraw from the study at any time without providing a reason for doing so. The characteristics of the participants are presented in Table 1.

**Table 1:** Anthropometric, physiological and performance characteristics of the 87 male and 36 female Norwegian cross-country skiers participating in this study (Mean ± SD).

**Variable**	**Male (n = 87)**	**Female (n = 36)**
Age, yrs^*^	22.8 ± 2.7	24.1 ± 4.5
Body height, m	1.83 ± 0.06	1.68 ± 0.05
Body mass, kg	75.5 ± 6.3	59.9 ± 2.4
Body mass index, kg·m^−2^	22.5 ± 3.1	21.2 ± 2.8
Maximum heart rate, beats·min^−1^	198 ± 10	196 ± 8
VO_2_max, L·min^−1^	5.71 ± 0.5	3.95 ± 0.5
VO_2_max, mL·min^−1^·kg^−1^	75.6 ± 4.7	65.8 ± 4.7
FIS points (distance)	95 ± 123	101.5 ± 59.8
FIS points (sprint)	93 ± 59	157 ± 105
Annual training volume, hours	666.5 ± 146.7	673.9 ± 146.2

* *Age of participants in the 2019–2020 season*.

*VO_2max_ – maximal oxygen uptake (highest self-reported value previous year). FIS, International Ski Federation*.

### Questionnaire

Data was collected via an online questionnaire (Nettskjema: https://nettskjema.no/) in which the athletes self-reported their anthropometric, physiological, and training characteristics, in addition to ski and pole length in classic and skating styles. The questionnaire was designed to take 7–10 min to complete and contained 13 questions: 12 questions asking for a numeric value and one open-ended question. A pilot study was organized among 40 skiers (19–23 years old men and women) to ensure that participants understood all questions. Based on feedback from this pilot, a minor revision was carried out to ensure valid information. The online questionnaire was distributed to 156 male and 71 female participants through Facebook Messenger, based on the result list from the National Championships 10 and 15 km individual time trials for women and men, respectively.

The participants were asked to report their pole and ski lengths for the classical and skating techniques according to the equipment's length description. It is also essential to notice that the competition organizers have regular controls for pole length violations (i.e., <83% of body height in classic), and the current study participants did not have any violations of this rule although many subjects reported classical pole lengths slightly above the 83% rule of the FIS (rule 343.8.2). This is caused by the way poles are measured: “from the bottom of the pole, and to the highest attachment on the strap,” while “body height is measured with ski boots on.” Thereby, there is a 3–4 cm difference in pole length between the length provided by the producer and the pole measurements taken to control for competition regulations. Confirmations from representants for the most important ski factories are the basis for the recommendations on ski length in this manuscript.

### Statistics

Questionnaire responses were summarized in numerical values to facilitate statistical analyses. Descriptive data for variables were recorded as means (SD. The Shapiro–Wilk test and standard visual inspection were used to examine the assumption of normality. To compare body-height-normalized pole and ski length between men and women, an independent samples *t*-test was used, while Pearson correlations were used to quantify the association between performance (FIS points), body-height-normalized pole and ski lengths. The threshold for interpretation of these correlations was: trivial (<0.1); small (0.1–0.3); moderate (0.3–0.5); high (0.5–0.7); very high (0.7–0.9); or practically perfect (0.9) (Calkins, 2005). The significance level was set at *p* ≤ 0.05 for all tests, and the analyses were carried out using SPSS Statistics v27 (SPSS Inc., Chicago, IL, USA).

## Results

**Figure 1:**
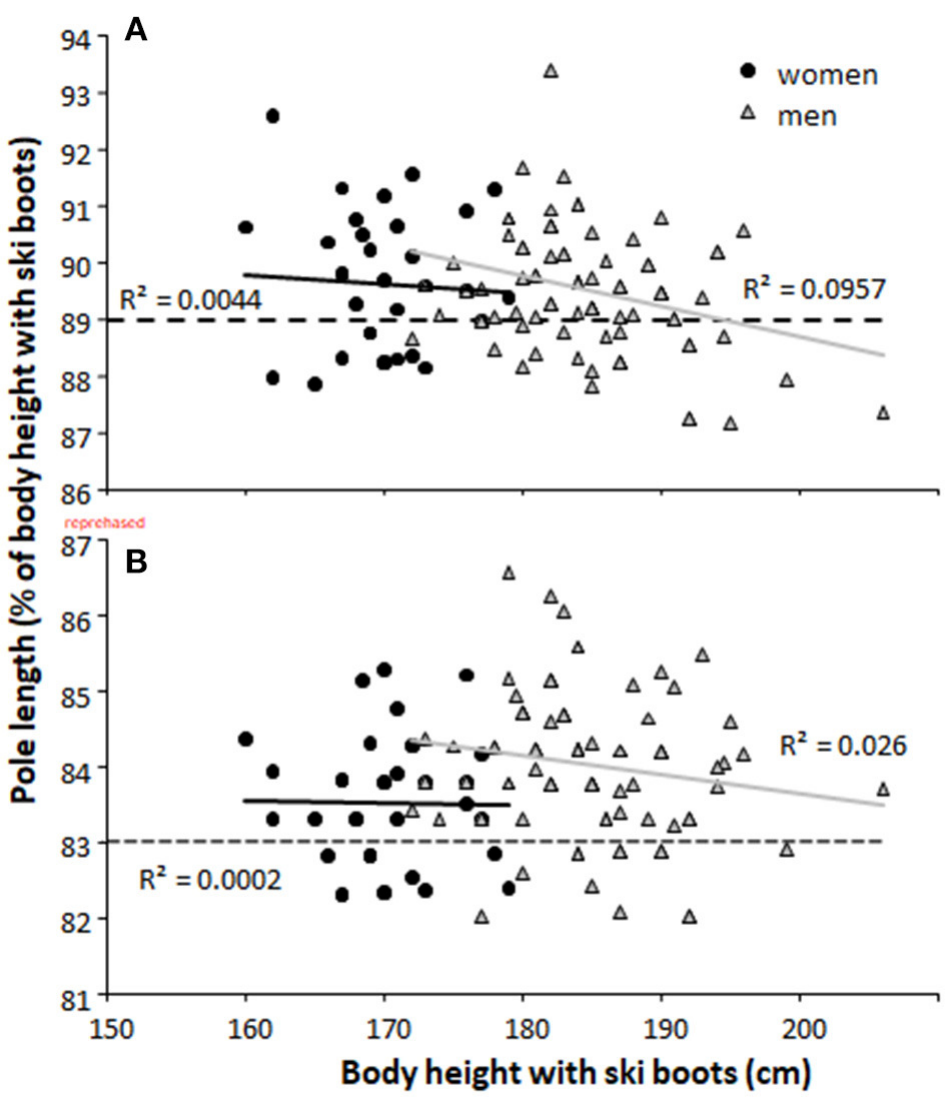
Pole length in percentage of body height (using ski boots) for men and women in the **(A)** skating and **(B)** classical cross-country skiing techniques. - - indicates recommendation of pole length (89%) of the ski factory = = indicates the limit of pole length (83%) according to the FIS rules in classical ski technique.

The body-height-normalized pole lengths used in the classical technique (Figure 1A) were on average 83.9 ± 0.9% for all athletes. Men (84.0 ± 0.9%) used significantly (*p* = 0.005) longer poles relative to their body height than women (83.5 ± 0.9%). In the skating technique (Figure 1B), the corresponding pole lengths were 89.5 ± 1.1%. No significant differences (*p* = 0.61) in body-height-normalized pole lengths in skating was found between men and women. No significant correlations were found between body-height-normalized pole length and body height (Figures 1A,B).

**Figure 2:**
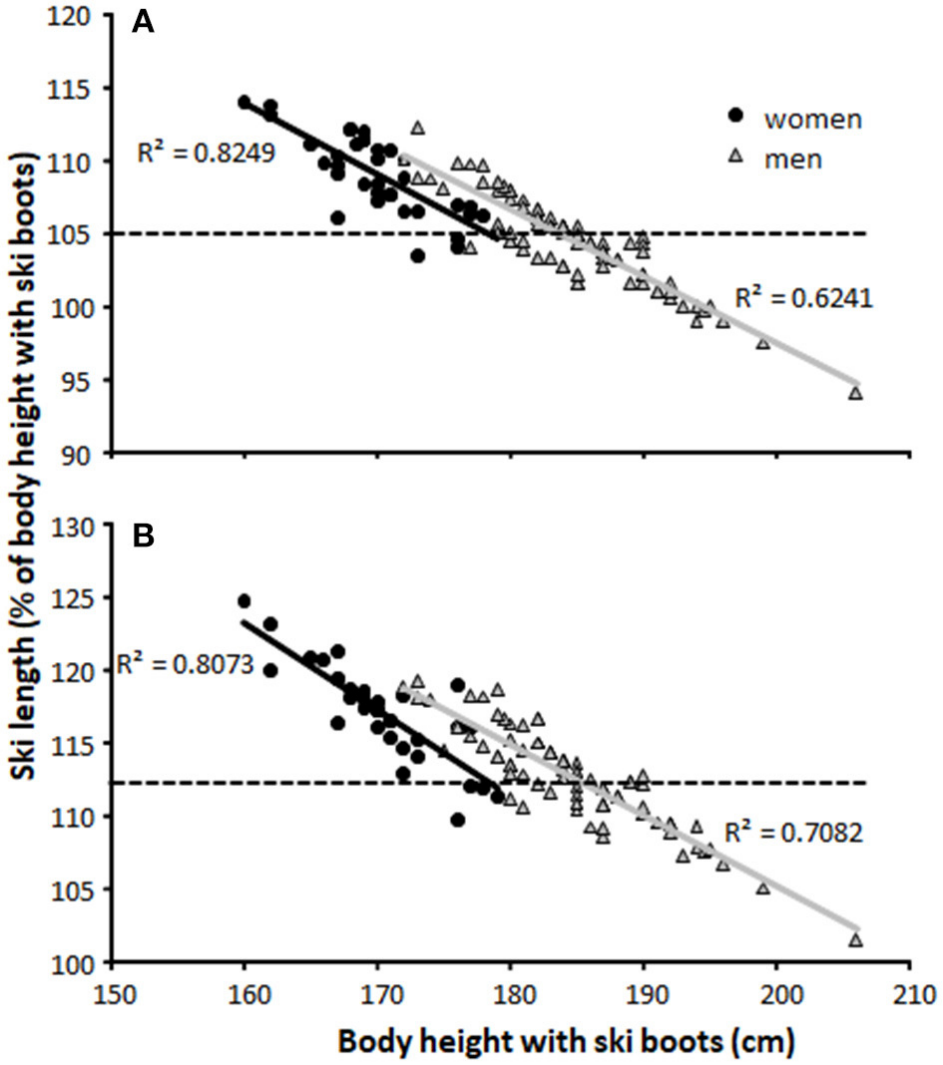
Ski length in relation to body height (using ski boots) for men and woman in the **(A)** skating and **(B)** classical cross-country skiing techniques. - - indicates recommendation of the ski factory.

For body-height-normalized ski lengths (Figures 2A,B), women (skating: 108.0 ± 2.8%, classical: 117.2 ± 3.2%) used significantly longer ski lengths than men (skating: 104.4 ± 3.0%, classical: 112.6 ± 3.3%). A very high to a practically perfect negative relationship was found between body-height-normalized ski length and body height (Figures 2A,B).

No significant correlation was found between body-height-normalized ski or pole lengths and sprint or distance FIS points (all *r* ≤ 0.17, all *p* ≥ 0.055) when all athletes' data were pooled. When analyzed within each gender, a moderate correlation between sprint FIS points and body-height-normalized pole length in both skating (*r* = 0.36, *p* = 0.030) and classical techniques (*r* = 0.43, *p* = 0.008) was found in women.

## Discussion

The purpose of this report was to document pole and ski lengths among elite male and female cross-country skiers in the classical and skating styles and to investigate sex and performance-level differences in body-height-normalized pole and ski lengths. The main findings were that: (1) most of the athletes used poles close to the length allowed by FIS in the classical style among both sexes, with men using slightly longer body-height-normalized poles than women; (2) body-height-normalized pole lengths in skating were similar in men and women, with the average pole length being approximately similar to that recommended by the industry (around 90% of body height); (3) women used relatively longer ski lengths than men in both styles, which was longer than recommended for women; and (4) only women showed moderate correlations between body-height-normalized pole lengths and performance (i.e., sprint FIS points), while no other correlations between ski and pole lengths and performance occurred.

Most of the skiers used poles close to the length allowed by the FIS in the classical style (83% of body height measured to the strap of the pole using ski boots) among both sexes, with men using slightly longer poles than women. The trend that national level skiers in Norway maximize their pole lengths in classic style underlines the importance of effective double-poling, where longer poles seem beneficial for classical skiing performance (Losnegard et al., 2017b). It appears that men are more aware of this advantage than women and have experienced that double-poling can be done more effectively with longer poles. However, the fact that there was a correlation between performance and pole length in female sprint skiing indicates that the best female sprint skiers also utilize this advantage.

Body-height-normalized pole lengths in skating were similar among men and women, with average lengths in line with the industry's recommendations (around 90% of body height). While 66 and 71% of the female and male skiers reported slightly longer poles than recommended (SWIX, 2020), 60% are within ± 1% of the recommended pole length and 99% within ± 2%. Accordingly, only 1% of skiers are using longer skating poles (92–94% of body height), which has been shown to have a potential benefit in previous research by improving work economy, treadmill and on-snow performance in the G3 sub-technique (Torvik et al., 2019, 2021b). However, the use of long poles has often been associated with the negative effect on skiing technique among coaches, such as adverse effects on the skiing rhythm, skiing with high shoulders, more tension in the muscles to lift the arms higher in the repositioning phase, slower repositioning of the poles, and increased air resistance. Whether these anecdotes really apply needs to be examined and systematic experimenting in both the classical and skating styles is required in order to find optimal lengths for individual skiers.

Women used relatively longer ski lengths than men in both styles, with women's skis being longer than typically recommended. Here, anecdotes from coaches and skiers are that longer skis glide better than shorter ones due to better weight distribution over a more extended nominal contact area, an advantage that is also confirmed in previous research (Breitschädel, 2012). Therefore, longer classical skis will be selected if they are soft enough to get sufficient grip. It is also known that the ski industry produces a smaller number of top skis for the smallest women, and female skiers as well as ambitious young boys and girls will therefore compete for the same pairs of skis within the recommended length for their body size. In such a case, choosing skis that are 5–10 cm longer enables higher number of top skis to choose from. This is also supported by communication with the ski industry, who argue that production of skis in different lengths is mainly dependent on financial reasonings (personal communication with Mobakken, 2021). In contrast, the tallest men are using skis close to the maximal ski length produced, since the longest classical and skating skis on the market are 207–210 and 190–195 cm, respectively. Accordingly, ~80% of the male cross-country skiers use the maximal ski lengths in both styles.

### Research Limitations and Future Recommendations

A main limitation of this investigation is the difference in pole length measurements defined by the FIS rules and the one used by the industry. This does not allow a direct comparison, but we have checked these differences for the most common pole types employed here. Along the same lines, normalizing for body height might not be an optimal procedure, since the anthropometric differences between athletes (e.g., differences in length of the head and the neck) may lead to differences in the rotation point of the shoulder between skiers with similar body height. In this context, the shoulder joint is the point of departure for transferring power from the body through the arms and to the poles. Previously, the shoulder height was a standard way to select both classical and skating poles (Bjerke, 2020), which still seems to be a more appropriate method than using body height. In future research, these aspects should be considered when discussing or analyzing pole lengths in cross-country skiing.

## Conclusion

This study reports pole and ski lengths chosen by elite male and female cross-country skiers and examines sex and performance-level differences in this respect. It seems clear that the best-performing male as well as female cross-country skiers use as long of classic ski poles as possible within the current regulations, which is likely to optimize their double-poling performance. In general, men tend to choose poles that are closer to this limit than women, which might be explained by the greater use of double-poling than diagonal stride in men's compared to women's classical competitions. However, longer body-height-normalized pole lengths among faster women in sprint indicate that faster women are able to better utilize the potential of using longer poles when double-poling. In skating, similar body-height-normalized pole lengths are used by men and women, with lengths similar to those recommended by the industry. For skis, women used relatively longer ski lengths than men in both styles, which were also longer than recommended. Whether this is due to longer skis being advantageous or a bias with more good skis produced with lengths optimal for men by the industry needs further examination. However, no significant correlations between ski length and performance were found, with close to perfect correlations between body height and ski length, indicating that ski length was purely chosen by body height within both sexes.

## Data Availability Statement

The dataset presented in this article may be obtained by contacting the corresponding author. Requests to access these datasets should be directed to Per-Øyvind Torvik, per.o.torvik@nord.no.

## Ethics Statement

Ethical review and approval was not required for the study on human participants in accordance with the local legislation and institutional requirements. The patients/participants provided their written informed consent to participate in this study.

## Author Contributions

P-ØT and ØS designed the study. P-ØT performed data collection and wrote the first draft of the manuscript. RT performed the statistical analyses. P-ØT, RT, and ØS contributed to interpretation of the results. RT and ØS contributed to write the final manuscript. All authors read and accepted the final version of the manuscript.

## Conflict of Interest

The authors declare that the research was conducted in the absence of any commercial or financial relationships that could be construed as a potential conflict of interest.
